# Clinical Characteristics and Management of Oral Candidiasis Associated with Calcinosis, Raynaud's Phenomenon, Esophageal Dysmotility, Sclerodactyly, and Telangiectasia Syndrome

**DOI:** 10.7759/cureus.76058

**Published:** 2024-12-20

**Authors:** Fu Sakai, Keita Takizawa, Akiko Okada-Ogawa, Kana Ozasa, Noboru Noma

**Affiliations:** 1 Department of Oral Medicine, Nihon University School of Dentistry, Tokyo, JPN

**Keywords:** anticentromere antibodies, antinuclear antibodies, crest syndrome, oral candidiasis, palmoplantar pustulosis

## Abstract

Calcinosis, Raynaud's phenomenon, esophageal dysmotility, sclerodactyly, and telangiectasia (CREST) syndrome, a systemic sclerosis subtype, features skin thickening, vascular issues, and organ involvement, causing complications in the gastrointestinal and musculoskeletal systems. Herein, we present a rare case of oral candidiasis, with CREST syndrome. The patient presented with xerostomia, tongue erythema, and burning pain. The patient reported finger stiffness, facial sclerosis, cold-induced pain, and a 10-year history of palmar-plantar pustulosis. Laboratory tests confirmed antinuclear antibodies (ANA) and anticentromere antibodies (1:1280), leading to a diagnosis of CREST syndrome. A fungal culture identified *Candida albicans*, and treatment with miconazole gel successfully resolved the infection. This case emphasizes the importance of recognizing oral manifestations in systemic autoimmune diseases, as conditions like xerostomia increase susceptibility to infections.

## Introduction

Calcinosis, Raynaud's phenomenon, esophageal dysmotility, sclerodactyly, and telangiectasia (CREST), a subtype of localized systemic sclerosis, represents an autoimmune connective tissue disorder characterized by calcinosis, Raynaud's phenomenon, esophageal dysmotility, sclerodactyly, and telangiectasia [1]. The condition leads to fibrosis of the skin and internal organs, with pronounced involvement of the esophagus and blood vessels. Oral manifestations include fibrosis of the oral mucosa, telangiectasia, and xerostomia, which results in decreased salivary flow, compromising oral defense mechanisms [2-4]. In patients with autoimmune diseases, oral candidiasis may develop due to immune dysfunction and mucosal changes [5,6]. Although reported cases remain limited, oral candidiasis in this context presents significant clinical relevance. This paper discusses a case of xerostomia and oral candidiasis associated with CREST syndrome, focusing on pathogenesis, clinical presentation, diagnostic approach, and therapeutic strategies, thereby underscoring the importance of oral manifestations in the early diagnosis and management of CREST syndrome.

## Case presentation

**Figure 1:**
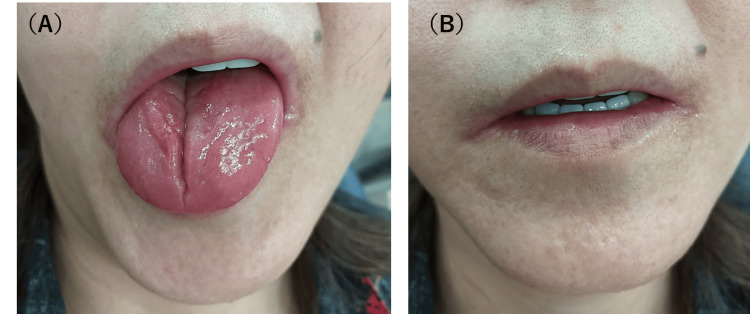
At the initial consultation, findings from the tongue and facial appearance Initial oral examination revealed atrophy of the tongue papillae and burning tongue pain due to xerostomia (A), along with erythema and angular cheilitis (B)

A 57-year-old female patient presented to the outpatient clinic at our hospital with complaints of dry mouth and tongue redness, which she had been experiencing for the past eight years. One month prior, she also reported tongue redness, burning pain, and difficulty with food intake. The patient described stiffness in her fingers, facial sclerosis, and changes and pain in her fingers due to cold exposure, with associated pallor. Her medical history includes palmar-plantar pustulosis, which was noted approximately 10 years ago. There were no other significant medical history or noteworthy findings. Clinical evaluation revealed atrophy of the lingual papillae, erythema, a central fissure, and angular cheilitis consistent with xerostomia (Figure 1).

**Figure 2:**
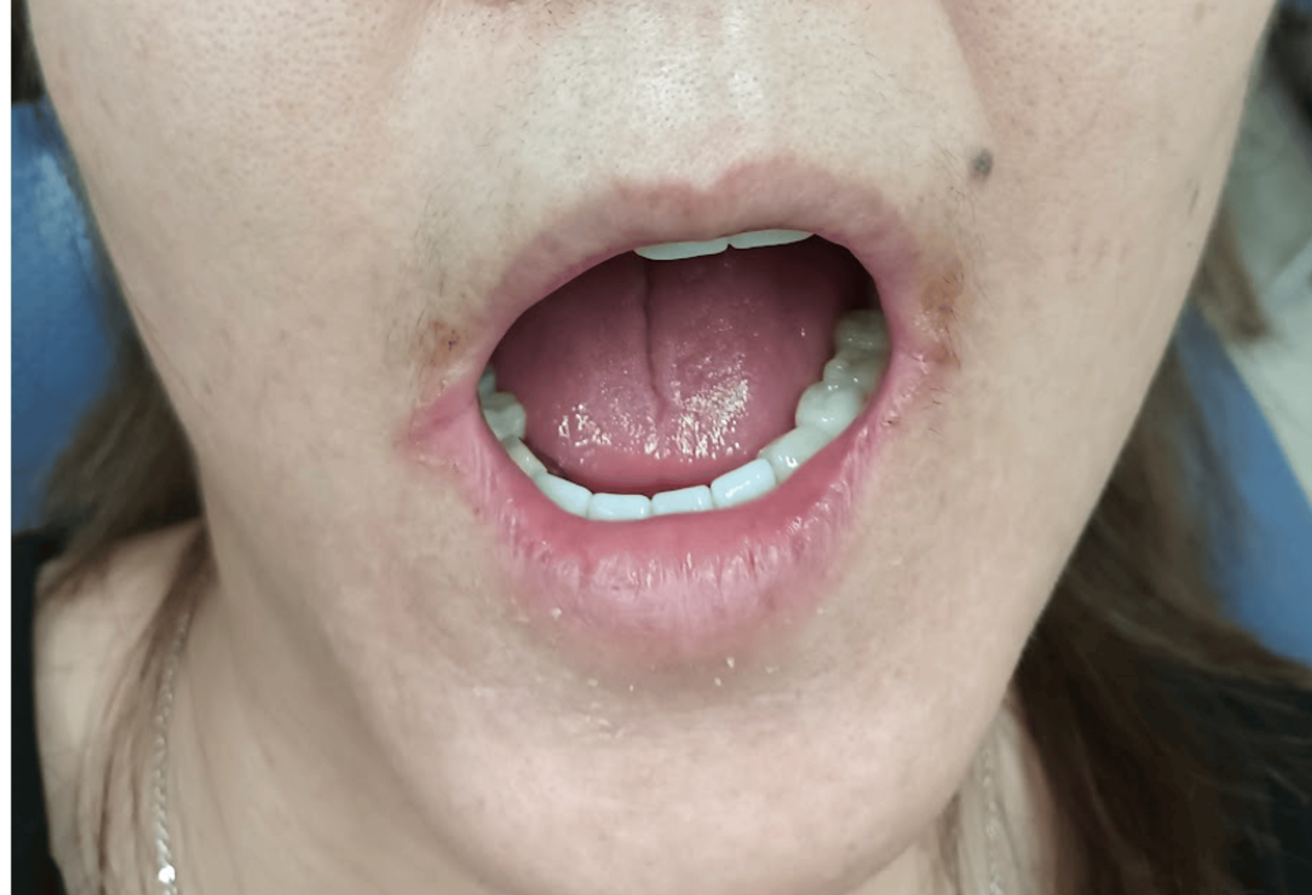
Findings of facial appearance during mouth opening Skin sclerosis was observed from the right side of the face to the lips, along with limited mouth opening

There was no palpable sclerosis of the lateral borders of the tongue or the floor of the mouth. The patient chewed a tasteless chewing gum, and the gum test result was 0.6 ml/10 minutes. The examination revealed fibrotic changes and rigidity of the facial skin, with significant restriction in the range of motion of the mouth. There were no signs of temporomandibular joint dysfunction, such as clicking, crepitus, or masticatory muscle pain; however, a right-sided deviation was observed during mouth opening (Figure 2).

**Figure 3:**
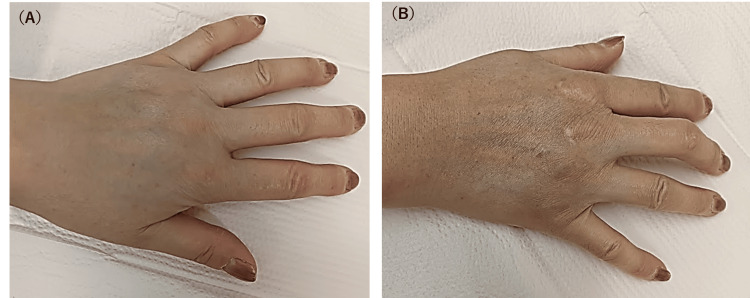
Findings of the wrist in the resting position and in the flexed position A: Clinical presentation showing skin sclerosis extending from the fingers to the palm. B: Mild flexion deformity of the fingers was observed

The skin sclerosis was distinctly evident from the fingers extending to the palms, with mild flexion contractures observed in the fingers (Figure 3).

**Figure 4:**
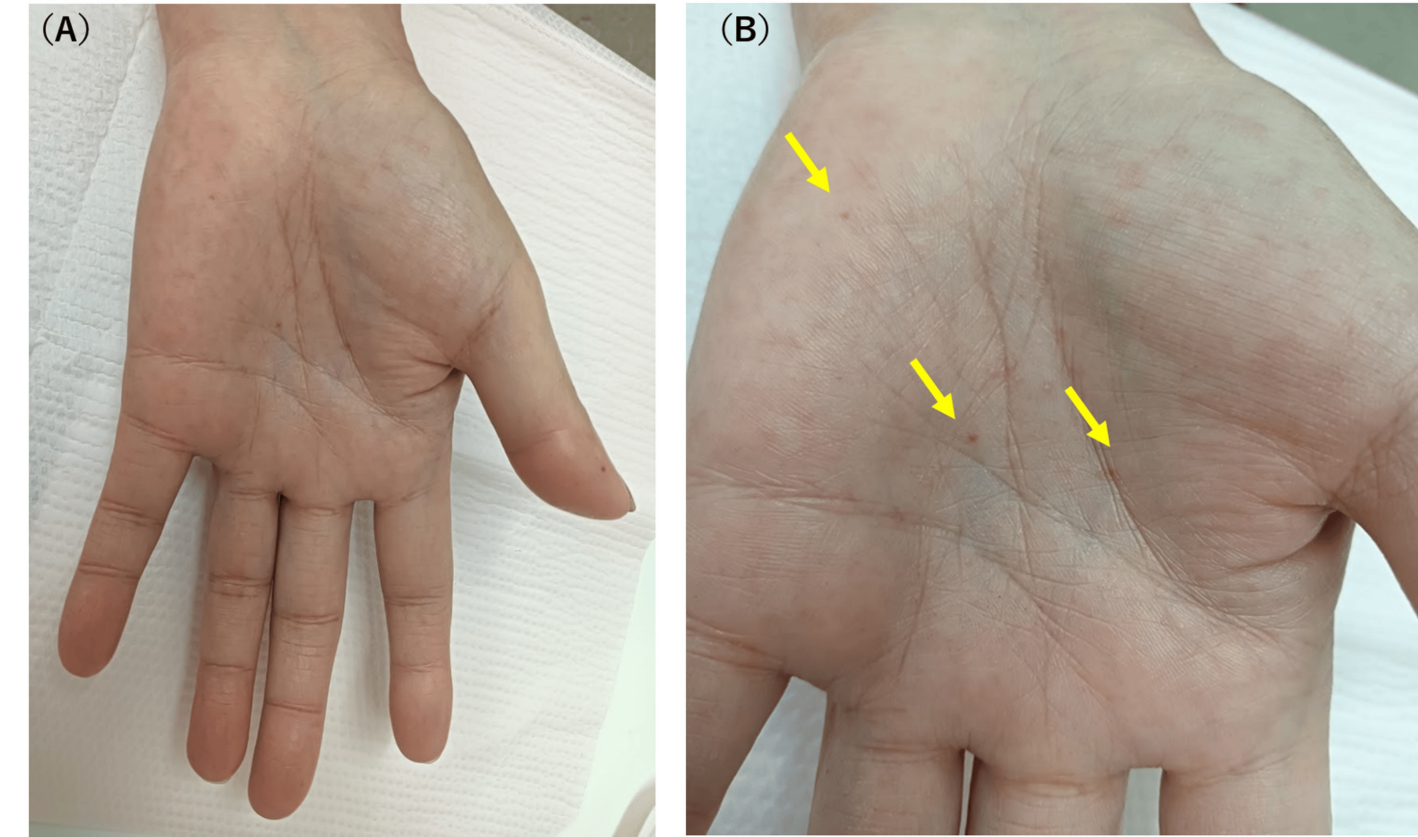
Findings of the palm A-B: Scattered petechiae were observed on the palm

Petechial hemorrhages were scattered across the palms. Small red macules were distributed, consistent with findings suggestive of capillary telangiectasia and involvement of small blood vessels (Figure 4).

**Figure 5:**
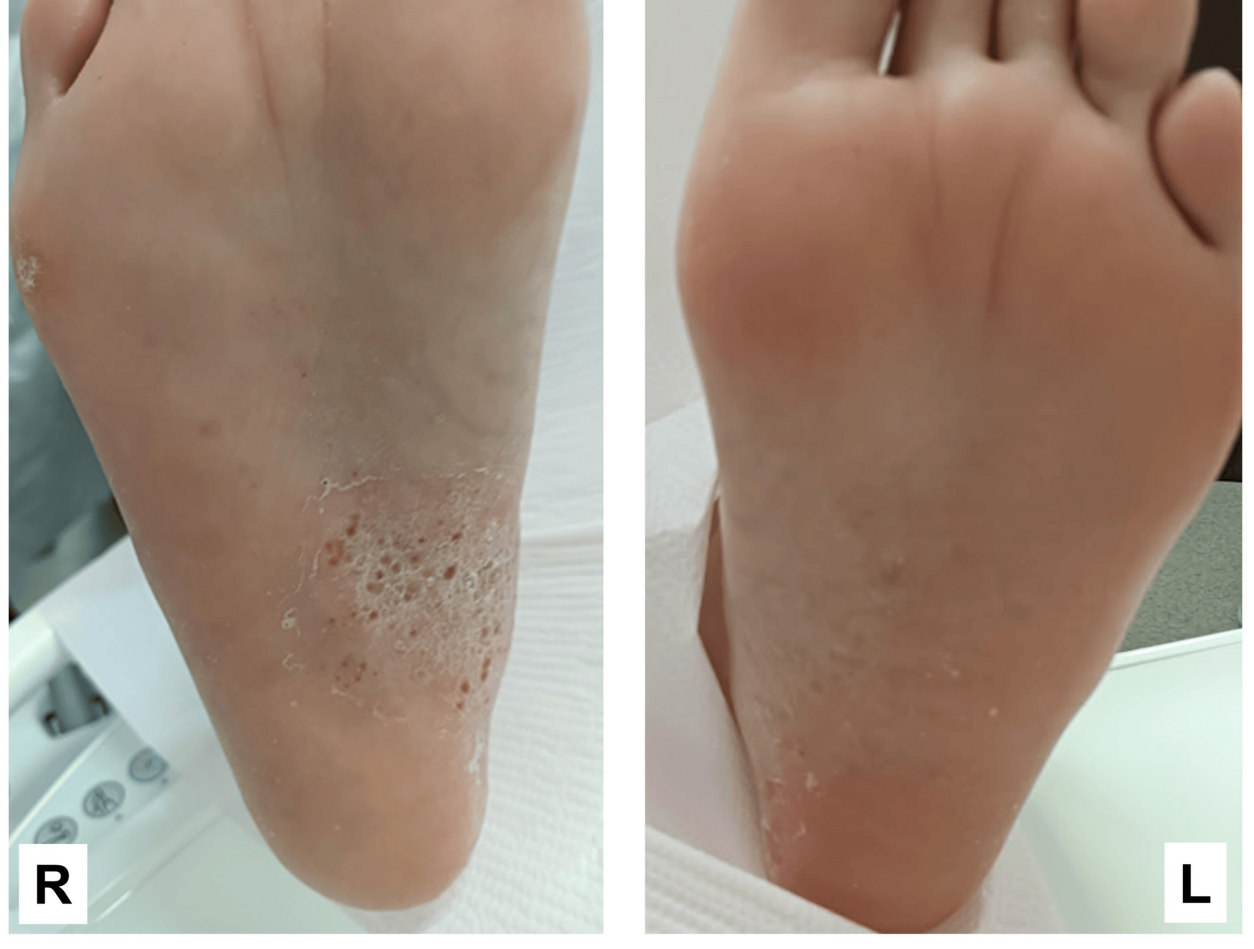
Findings of the sole R: right; L: left While the symptoms of palmoplantar pustulosis were more prominent on the right side, they also appeared on the left foot

Additionally, pustular lesions resembling palmar-plantar pustulosis were observed on the right sole (Figure 5).

Laboratory investigations by the rheumatology department showed positive results for antinuclear antibodies (ANA) and anticentromere antibodies (1:1280) (Table 1).

**Table 1:** Results of laboratory investigations by the rheumatology department RBC: red blood cells; WBC: white blood cells; PLT: platelets; HGB: hemoglobin; anti-SSA antibodies: anti-Sjögren's syndrome A antibodies; anti-SSB antibodies: anti-Sjögren's syndrome B antibodies; antinuclear antibodies: autoantibodies targeting nuclear components; anticentromere antibodies: antibodies against centromere proteins; RF: rheumatoid factor; CRP: C-reactive protein Laboratory investigations showed elevated levels of antinuclear antibodies, anticentromere antibodies, and rheumatoid factor

Laboratory parameters with units	Patient values	Reference range
RBC (104/μL)	445	353-466
WBC (103/μL)	10.0	3.0-7.8
PLT (104/μL)	12.9	13.8-30.9
HGB (g/dL)	13.6	10.6-14.4
Anti-SSA antibodies	Negative	0‐0.9
Anti-SSB antibodies	Negative	0‐0.9
Antinuclear antibodies	1280	0‐40
Homogeneous	(-)	0‐40
Speckled	(-)	0‐40
Nucleolar	(-)	0‐40
Peripheral	(-)	0‐40
Anticentromere antibodies	1280	0‐40
Cytoplasmic	(-)	0‐40
Rheumatoid factor (RF)	29	0‐15
CRP (mg/dL)	0.95	≦0.3

Based on these serological findings and clinical criteria, the patient was diagnosed with CREST syndrome. At the initial consultation, a bacterial culture was performed by swabbing the dorsum of the tongue, which isolated *Candida albicans*. The patient was treated orally with miconazole (Fluconazole Gel) for seven days (administered four times daily), which led to the resolution of the lingual burning sensation and erythema. The patient was informed by the rheumatology department that Sjögren's syndrome was excluded from the differential diagnosis and that no interventions, including steroid therapy aimed at halting disease progression, would be undertaken. Currently, the patient is being monitored in the oral medicine department, where management of xerostomia is being approached through measures such as saliva substitutes, oral hygiene optimization, and adjunctive therapies including massage and stretching exercises.

## Discussion

The acronym "CREST" is derived from the initial letters of the clinical symptoms: calcinosis cutis, Raynaud's phenomenon, esophageal dysmotility, sclerodactyly, and telangiectasia. According to the American College of Rheumatology and the European League Against Rheumatism, CREST syndrome is considered a form of systemic sclerosis that meets at least three of the five clinical criteria [7,8]. According to our collected data, there have been a total of eight reported cases of oral and maxillofacial diseases associated with CREST syndrome, including our cases (Table 2) [2,3,4,7,9,10].

**Table 2:** Report of jaw and oral diseases associated with CREST syndrome CREST: calcinosis, Raynaud's phenomenon, esophageal dysmotility, sclerodactyly, and telangiectasia This report examines the clinical findings from seven cases, categorizing the associated oral symptoms and their prevalence. While the patients had different underlying conditions and specific oral lesions, all showed distinctive symptoms related to the oral mucosa and gingiva

Author	Age	Gender	Disease duration	Systemic manifestation	Oral mucosa involvement	Final diagnosis	Outcome
Stanford et al. [9]	38	Female	9 years	She exhibited Raynaud's phenomenon, esophageal dysmotility, telangiectasia, and calcinosis in one finger, and her phlebotomists noted "hardened" skin	Telangiectasia was observed on the lips, tongue, and gingiva. Some of the oral lesions failed to blanch under the pressure of a glass slide and thus resembled angiomata	CREST syndrome. Laboratory tests showed antinuclear antibodies (ANA) to be positive with a high titer at 1:640 and an anticentromere pattern	No data
Lauritano et al. [4]	72	Female	No data	Raynaud's phenomenon. The extraoral examination showed hardened and stiff skin, pale to reddish irregular macules across the face, along with telangiectasias and acrocyanosis	The patient experienced oral pain and difficulty with swallowing and eating, compounded by denture instability. Examination revealed tongue stiffness and speckled red and white lesions on the hard palate and vestibule. A diagnosis of Sjögren's syndrome was also confirmed	CREST syndrome. No data	Management of Sjögren's syndrome includes saliva stimulants, substitutes, and dental care to prevent xerostomia-related issues like caries, with a focus on oral hygiene and regular dental check-ups
Dixit et al. [10]	20	Male	2 years	There were limited, well-defined, oval-shaped hyperpigmented lesions on the abdomen and back, with a fibrotic texture upon palpation. Examination of the head and neck showed significant asymmetry, with noticeable atrophy of fat, muscle, and subcutaneous tissue on the left side of the face, giving it a shrunken appearance. The skin on the affected side appeared sclerotic	The left side of the tongue was atrophied and rigid, while the other oral mucosa appeared normally moist	CREST syndrome. Serological tests were positive for double-stranded DNA and antimitochondrial antibodies. A skin biopsy indicated a thickened epidermis and reticular dermis with closely packed collagen bundles, suggesting morphea	No data
Dixit et al. [10]	53	Female	7 years	The patient's face appeared expressionless. On palpation, the skin around the perioral region was sclerotic. Her fingers showed pale, indurated skin, which worsened with cold exposure, indicating Raynaud’s phenomenon. Additionally, telangiectasia was present on her fingers	The patient had microstomia caused by rigid perioral skin, along with xerostomia and an erythematous patch on the palate	CREST syndrome. Serological reports showed elevated serum C3 levels, and the RNA profile was positive for centromere B, Scl-70, and Ro-52. A hand-and-wrist radiograph revealed acro-osteolysis of the terminal phalanges in the middle finger of the right hand	No data
Arana-Guajardo et al. [7]	54	Female	2 years	The patient presented with dyspepsia and intermittent dysphagia to solids, along with arthralgia in the hands and wrists. The skin exhibited telangiectasia	Palatal telangiectasias	CREST syndrome. There were positive ANA antibodies (1:1280) with a centromeric pattern and positive anticentromere antibodies (1:10240)	No data
Bunn et al. [3]	42	Female	Phenomenon which was initially diagnosed in her early teens	The facial skin around the mouth exhibited multiple asymptomatic telangiectases. The patient presented with Raynaud's phenomenon, sclerodactyly, and localized areas of calcinosis cutis (calcium deposits in the skin). Additionally, the patient reported experiencing mild dysphagia and gastroesophageal reflux	Gingival recession was observed on teeth numbers 41, 43, 45, 31, 34, and 13. There were also multiple asymptomatic telangiectases present on the palate and tongue	Laboratory testing showed positive ANA and anticentromeric antibodies, leading to a rheumatologist diagnosing CREST syndrome based on the clinical features	Nonsteroidal anti-inflammatory drugs for digital and joint pain
Gilligan et al. [2]	26	Female	15 years	Dermatological and nail alterations were observed, including sclerodactyly, which caused the fingers to bend due to skin tightening. The right elbow exhibited notable wrinkles, cracked, rigid, dry skin, and scabby areas. An extraoral examination revealed skin rigidity, cracking, and facial sclerosis, with a hardened, sclerotic, hypochromic appearance and pallor of the lip vermilion	The mucosa was tense and rigid, complicating the clinical examination. The lateral borders of the tongue were pale and hard to the touch, and the tongue exhibited rigidity indicative of ankyloglossia. The buccal mucosa presented similar characteristics to the affected areas of the oral mucosa	CREST syndrome. The histological findings suggested Sjögren's syndrome (SS), while the anti-SD 70 antibody was negative. Based on the analyzed clinical criteria, the patient met the criteria for CREST syndrome	No data
Our case (Sakai et al.)	57	Female	8 years	Mild flexion contractures of the fingers were observed. Pustular lesions resembling palmar-plantar pustulosis appeared on the right sole, accompanied by scattered petechiae on the palms. Small red macules were distributed, indicating capillary telangiectasia and small blood vessel involvement	Clinical evaluation showed atrophy of the lingual papillae, erythema, and angular cheilitis, which are consistent with xerostomia. There was no palpable sclerosis observed in the lateral borders of the tongue or the floor of the mouth	CREST syndrome. Laboratory investigations by the rheumatology department revealed positive antinuclear antibodies (ANA) and positive anticentromere antibodies(1:1280)	The patient was treated orally with miconazole (Fluconazole Gel) for 7 days, administered four times daily, resulting in the resolution of the lingual burning sensation and erythema

The average age of the patients was 45.3 years, with seven cases of female patients and one case of a male patient. The average duration of illness or the time until diagnosis (CREST syndrome) was 10.4 years. This report analyzes the clinical findings from seven cases, organizing the associated oral symptoms and their frequency. Although the patients presented with various underlying conditions and specific oral lesions, all exhibited characteristic symptoms involving the oral mucosa and gingiva. Based on these data, clinical insights are drawn. The most frequently observed symptoms in these seven cases were as follows: telangiectasia in four cases, xerostomia and tongue stiffness in three cases, gingival recession in one case, and denture instability with oral pain in one case. Systemic manifestation included Raynaud's phenomenon in four cases, telangiectasia in five cases, skin thickening in six cases, sclerodactyly in four cases, intermittent swallowing difficulties in two cases, esophageal motility disorders in one case, calcium deposits in the skin in two cases, and notable wrinkles and cracking of the skin in two cases.

To the best of our knowledge, this is the first reported case of oral candidiasis associated with CREST syndrome. Our case meets three of the clinical criteria of CREST syndrome, including Raynaud’s phenomenon, sclerodactyly, and telangiectasia, based on both systemic and oral findings. The clinical features and blood test results, including positive anticentromere antibodies, led to the diagnosis of CREST syndrome complicated by xerostomia and oral candidiasis.

Patients with CREST syndrome often exhibit sclerotic changes in the oral mucosa and the soft tissues around the mouth. Gilligan et al. have noted that patients frequently report limited mouth opening and tongue stiffness, leading to difficulties with speech and swallowing [2]. These functional impairments are attributed to mucosal rigidity and whitish fibrosis observed in the soft palate and uvula. In this case, while no rigidity was observed in the tongue or oral mucosa, sclerotic changes were noted on the right facial skin, causing a deviation to the right during mouth opening. Additionally, there were no signs of temporomandibular joint dysfunction (such as clicking, crepitus, or masticatory muscle pain). In the differential diagnosis of CREST syndrome, mouth-opening limitation due to skin sclerosis is a key point to consider.

Previous studies have shown that patients with CREST syndrome may present with periodontal disease characterized by mucogingival lesions, such as the loss of attached gingiva and multiple gingival recessions [3,9]. CREST syndrome may also be associated with xerostomia due to overlapping autoimmune phenomena with Sjögren’s syndrome [11,12]. In this case, stiffness and flexion deformities of the fingers are often observed. As a result, it becomes difficult for them to grip a toothbrush firmly and manipulate it effectively, which hinders proper oral cleaning and leads to inadequate oral hygiene. Additionally, the presence of xerostomia likely contributed to the secondary development of oral candidiasis. The patient had been aware of palmoplantar pustulosis (PPP) for 10 years and developed xerostomia eight years ago, possibly in connection with CREST syndrome.

PPP has been associated with autoimmune diseases [13]. PPP is characterized by recurrent pustules and vesicles on the palms and soles, and its pathogenesis is suggested to involve autoimmune mechanisms. Specifically, it is believed that PPP arises from aberrant immune responses, with both innate and adaptive immunity playing a role in its development. PPP is also known to coexist with other autoimmune diseases, such as psoriasis, rheumatoid arthritis, and Crohn's disease [13]. These diseases may share common immunopathological mechanisms with PPP, which could explain the concurrent manifestation of multiple autoimmune disorders. T cells and B cells, key immune cells, are thought to be involved in the pathogenesis of PPP [14]. This involvement is similar to the role that these immune cells play in other autoimmune diseases, further supporting the hypothesis that PPP is closely related to autoimmune processes.

However, there have been no previous reports in the literature linking these conditions. While it is unclear whether CREST syndrome and palmoplantar pustulosis developed independently or have a mutual relationship, this case raises an important issue that warrants further investigation.

## Conclusions

This case highlights the rare association of oral candidiasis with CREST syndrome, emphasizing the importance of early recognition and management of oral manifestations in autoimmune connective tissue disorders. The presence of xerostomia, sclerotic changes, and oral candidiasis in CREST syndrome underscores the need for comprehensive oral care, including maintaining oral hygiene, and interdisciplinary collaboration between oral medicine and rheumatology. Additionally, this case introduces the possibility of an unexplored connection between CREST syndrome and palmoplantar pustulosis, raising important questions about potential pathophysiological relationships that warrant further study.
